# Super-resolution imaging reveals the sub-diffraction phenotype of Zellweger Syndrome ghosts and wild-type peroxisomes

**DOI:** 10.1038/s41598-018-24119-2

**Published:** 2018-05-17

**Authors:** Kareem Soliman, Fabian Göttfert, Hendrik Rosewich, Sven Thoms, Jutta Gärtner

**Affiliations:** 1 Department of Pediatrics and Adolescent Medicine, University Medical Center Göttingen, Georg August University Göttingen, Robert-Koch-Strasse 40, 37075 Göttingen, Germany; 2 Department of NanoBiophotonics, Max Planck Institute for Biophysical Chemistry, Am Faßberg 11, 37077 Göttingen, Germany; 3 Present Address: Optical Nanoscopy, Laser-Laboratorium Göttingen e.V., 37077 Göttingen, Germany

## Abstract

Peroxisomes are ubiquitous cell organelles involved in many metabolic and signaling functions. Their assembly requires peroxins, encoded by *PEX* genes. Mutations in *PEX* genes are the cause of Zellweger Syndrome spectrum (ZSS), a heterogeneous group of peroxisomal biogenesis disorders (PBD). The size and morphological features of peroxisomes are below the diffraction limit of light, which makes them attractive for super-resolution imaging. We applied Stimulated Emission Depletion (STED) microscopy to study the morphology of human peroxisomes and peroxisomal protein localization in human controls and ZSS patients. We defined the peroxisome morphology in healthy skin fibroblasts and the sub-diffraction phenotype of residual peroxisomal structures (‘ghosts’) in ZSS patients that revealed a relation between mutation severity and clinical phenotype. Further, we investigated the 70 kDa peroxisomal membrane protein (PMP70) abundance in relationship to the ZSS sub-diffraction phenotype. This work improves the morphological definition of peroxisomes. It expands current knowledge about peroxisome biogenesis and ZSS pathoethiology to the sub-diffraction phenotype including key peroxins and the characteristics of ghost peroxisomes.

## Introduction

Peroxisomes are single-membrane bounded eukaryotic organelles that carry out important metabolic as well as signaling functions in virtually all tissues. Malfunction of peroxisomes is associated with peroxisome-specific diseases and contributes to an increasing range of human pathology^1–6^. In conventional fluorescence microscopy, peroxisomes show a round or tubulated morphology. Size and distribution depend on the metabolic and signaling requirements of the cell^7^. In mammalian cells, the diameter of peroxisomes ranges between 50 and 200 nm, which lies below the diffraction limit for the resolution of optical microscopy^8,9^. Today, super-resolution microscopy allows the imaging of intracellular compartments below the diffraction limit, also referred to as sub-diffraction imaging. Peroxisome biogenesis requires peroxins, which are encoded by *PEX* genes^10^. To date, there are 16 known human peroxins and 35 altogether in all species^7,11^. The majority of peroxins are integral peroxisomal membrane proteins (PMPs). The early membrane peroxins (PEX3, PEX19, and PEX16) are essential for precise peroxisomal localization of the other PMPs^12^. In the absence of these peroxins, PMPs are generally mistargeted and destabilized. A special subset of the peroxins is involved in translocation of peroxisomal matrix proteins across the peroxisome membrane^13^. The AAA-type ATPase peroxins PEX1 and PEX6 are involved in recycling of PEX5 or PEX7, the receptors for PTS1 and PTS2 matrix proteins, respectively. The peroxisome translocation assembly consists of two main complexes: a docking/translocation complex (PEX13 and PEX14) and a RING complex (PEX2, PEX10 and PEX12). PEX2 and PEX12 act as E3 ubiquitin ligases essential for PEX5 ubiquitylation in receptor recycling, and PEX2 was recently shown to be involved in the ubiquitylation of PEX5 and PMP70 targeting peroxisomes for degradation by pexophagy^14^. The endoplasmic reticulum (ER) was found to be essential for peroxisome membrane protein sorting and early membrane biogenesis^15–18^. In yeast, it was shown that formation of the mature translocon requires the PEX1- and PEX6-dependent heterotypic fusion of two ER derived pre-peroxisomal vesicles (PPVs) populations carrying different peroxins^19^. However, more recent results suggest that the docking and RING subcomplexes can localize on the same membrane, and that the apparent non-colocalization is a result of enhanced autophagy found in *pex*1 and *pex6* mutant cells^20,21^. Moreover, the ghost peroxisome structures, i.e. membranes remnants with nearly empty matrix, in *pex*1 and *pex6* knockout yeast cells were shown to harbor docking/translocation complex and RING proteins, while reintroduction of the defective AAA peroxin only restored peroxisome import of matrix proteins^22^.

Peroxisome proliferation requires, among others, PEX11-type peroxisomal proliferators and dynamin-related fission factors^23,24^. The overexpression of PEX11β alone leads to hyper-tubulated peroxisomes^25^. It is not known, however, if PEX11β plays a role in the constriction of proliferating peroxisomal membranes *in vivo*^26^. Dynamin Like Protein-1 (DLP1) and Mitochondria Fission Factor (MFF) are shared between mitochondria and peroxisomes^27–29^. DLP1 forms helical rings on mitochondrial constrictions^30^, however, their structural arrangement at peroxisomes is unclear.

Mutations in any of the human *PEX* genes are associated with peroxisome biogenesis disorders (PBDs), with Zellweger syndrome (ZS) being the most severe and most prevalent within the Zellweger syndrome spectrum (ZSS)^1,31^. ZSS consists of more than fourteen complementation groups, each caused by mutation of one *PEX* gene^32^. We have a longstanding interest in the diagnostic process and in developing treatment options for ZSS patients. After the clinical evaluation and the routine metabolic work-up including the measurement of very-long-chain fatty acids (VLCFA), phytanic and pristanic acid, di- and trihydroxycholestanoic acid in blood of the patients as well as plasmalogens in erythrocyte lipids, we use a cell-based assay to determine the *PEX* gene affected in these patients^33^. In the context of this diagnostic process, we collected more than 200 different ZSS patient fibroblast cell lines. Cultivated primary human fibroblasts of these patients served as the basis for this study.

Ghost peroxisomes are the cellular hallmark of most ZSS cells. Ghost peroxisome membrane structures stain positive for PMPs and, depending on the defective *PEX* gene and type of mutation, they show little or no matrix content^34,35^. The peroxisomal ghosts have broad distribution of size and abundance^36^. Their sizes vary among the different complementation groups and they vary depending on the type of mutation within the same complementation group^36–38^. Studies conducted in patient fibroblasts and genetically perturbed yeast have found that mutations in the AAA peroxins and docking factors show large ghost structures, while RING factor mutations exhibited smaller ghosts and more of normal-sized peroxisome structures^36,39^. In contrast, clinical complementation groups that affect early peroxins, such as PEX3 and PEX19, exhibit mislocalized PMPs that are immediately degraded in the cytosol with little or no sign of peroxisomal ghost remnants^40^. Further, varying levels in the protein abundance of PMP70 have been observed between different ZS patients^41,42^, but the underlying mechanism behind protein abundance variabilities and whether the protein abundance plays a direct role in ghost size heterogeneity remains elusive.

In this study, we used super-resolution microscopy to investigate in detail the sub-diffraction structure of human peroxisomes in health and ZSS patients.

## Results

### Optical nanoscopy of human peroxisomes

**Figure 1:**
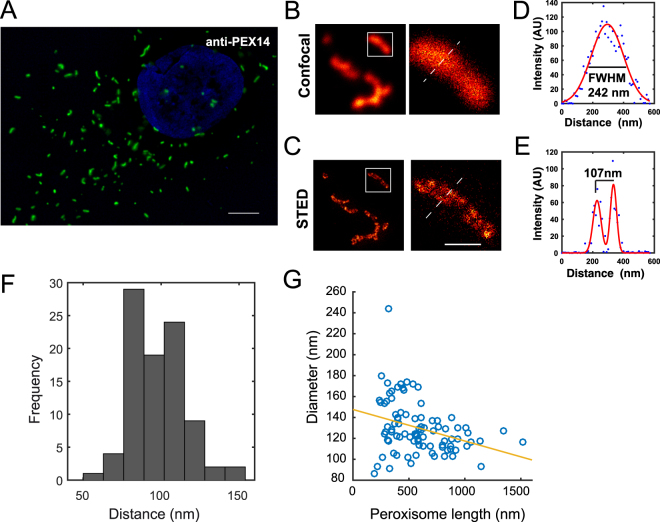
STED nanoscopy reveals peroxisome membrane and lumen. (**A**) Widefield image of human skin fibroblast peroxisomes probed with polyclonal rabbit anti-PEX14 antibody and labeled with secondary antibodies coupled to the Atto594 dye, which is spectrally compatible with the widefield filter sets. Scale bar = 5 µm. (**B**,**C**) Confocal and STED image of the same peroxisomal structure. Scale bar 200 nm. (**D**,**E**) Line scan analysis (dashed lines). (**F**) Histogram of sub-diffraction diameter of peroxisomes immunostained with anti-PEX14 antibodies and labeled with KK114 conjugated to secondary antibody measured by distance from two maxima of two component Gaussian fit. Peroxisome mean diameter (d_mean_) = 98.1 ± 17.1 (±SD). N = 90 peroxisomes from five randomly chosen cells. (**G**) Pearson correlation of peroxisome diameter and peroxisome length measured in nm. Peroxisome straightened structures thickness was measured by calculating the Full Width Half Maximum (FWHM). The data are representative of at least 10 independent cells. The data show significant inverse relationship at a p < 0.05.

Peroxisomes are bounded by a single membrane bilayer surrounding its luminal matrix. In diffraction-limited light microscopy, human peroxisomes appear as spherical or elongated structures distributed across the cytoplasmic cell volume (Fig. 1A). Thus, we employed Stimulated Emission Depletion (STED) microscopy, which offers diffraction-unlimited resolution, to analyze the size and structure of peroxisomes. We stained the peroxisomal membrane of human skin fibroblasts with anti-PEX14 antibodies and coupled secondary antibodies for analysis by a 775 nm STED microscope. STED imaging revealed the peroxisomal membrane and the lumen with a resolution below 50 nm (Fig. 1B,C). In confocal microscopy, the estimated diameter of a peroxisome appears to be ~250 nm, measured by full width at half maximum (FWHM) of the Gaussian fitted line scan (Fig. 1D). This corresponds to the resolution limit of the microscope and can therefore only pose an upper limit for its size. Imaging the same position of the peroxisome by super-resolution microscopy, the diameter can now be determined to be ~100 nm, as measured by the distance between the peaks of the Gaussian fitted line scan (Fig. 1E). We quantified the diameter of 90 peroxisomes and found a mean peroxisomal diameter of 98.1 ± 17.1 (±SD) (Fig. 1F), which is smaller than the peroxisome size previously measured by super-resolution microscopy^43^. We found that elongated peroxisomes tend to show a comparably smaller diameter on the short axis, but all the peroxisomes observed here confirmed a smaller diameter below the diffraction limit of conventional light microscopy (Fig. 1G). The majority of peroxisomes show a length of more than 250 nm and the apparent length of peroxisomes does not change with STED imaging because the increase in resolution has a more drastic effect on the structures below the limits of resolution, i.e. the diameter (short axis) of the peroxisome as opposed to the length (long axis). Therefore, elongated peroxisomes may look more spherical in widefield microscopy owing to resolution and diffraction limit effect rather than their true morphology. Altogether, our data show that the nanometer resolution offered by STED imaging is essential to reveal peroxisome structural details under physiological conditions by fluorescence microscopy.

**Figure 2:**
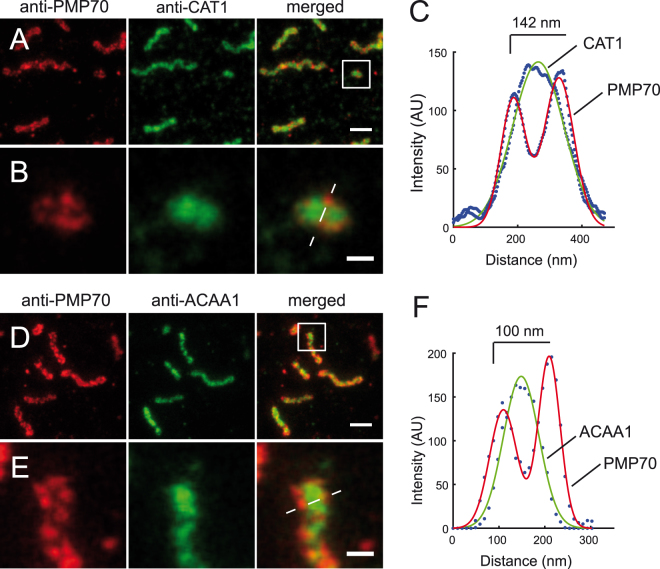
Two-color STED nanoscopy of peroxisome membrane and matrix. Dual immunofluorescence on human skin fibroblasts. (**A**) Monoclonal anti-PMP70 labeled with KK114 secondary label in red, polyclonal rabbit anti-Catalase (anti-CAT1) labeled with Atto594-coupled secondary in green, and a merge of both (right). (**B**) Blow-up of box in (**A**). (**C**) Gaussian fit of the line scan marked in (**B**). (**D**) Anti-PMP70 labeled with KK114-coupled secondary antibody in red, polyclonal rabbit anti-acetyl-CoA acyltransferase1 (anti-ACAA1) in green, and merged images of both. (**E**) Inset magnification of (**D**). (**F**) Gaussian fit of the line scan marked in (**E**). Images were smoothed by 3 × 3 average filter and linearly scaled. Scale bars 500 nm (**A**,**D**), and 100 nm (**B**,**E**).

**Figure 3:**
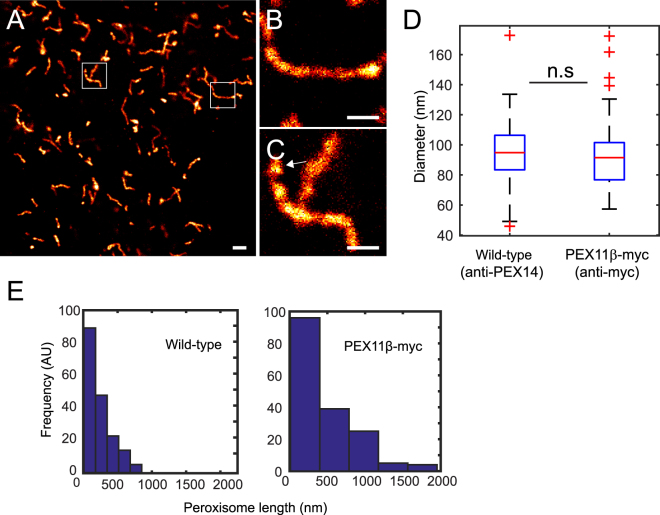
STED sub-diffraction image of hyper-tubulated peroxisomes. Peroxisome proliferation was induced by overexpression of PEX11β. (**A**) STED overview image of HeLa cell overexpressing PEX11β-myc fusion 24 hours after transfection, probed with a monoclonal anti-myc antibody and labeled with a secondary antibody conjugated to KK114 dye. (**B**,**C**) Blow up of hyper-tubulated PEX11β-myc structures. Arrow indicates a vesicle that may be budding of from the peroxisome. (**D**) Size (diameter) distribution of wild-type and hyper-tubulated peroxisomes. PEX11β-myc tubule’s diameter (probed with anti-Myc) was measured by FWHM of the Gaussian fit (N = 200 line scans from 70 peroxisomes from five cells). The mean diameter (d_mean_) = 94.9 ± 21.8 nm for wild-type and (d_mean_) = 91.8 nm ± 20.6 nm (±SD) for PEX11β-myc. For comparison, untransfected HeLa cells probed with anti-PEX14, quantified by distance between peaks of Gaussian fit membrane profiles (N = 74 profiles from 16 independent slices). Differences in the peroxisomal mean diameter were not significant (n.s) with p > 0.05. Scale bars 500 nm. (**E**) Histogram of peroxisome length showing PEX11β-myc hyper-tubulation >800 nm (N = 169 peroxisomes). Control: HeLa cells probed with the anti-PEX14 show no (N = 142 peroxisomes).

In order to visualize the distribution of endogenous peroxisome matrix proteins together with the peroxisomal membrane, we used two-color STED imaging on peroxisomal membranes labeled by anti-PMP70 together with anti-catalase (CAT1) or anti-acetyl-CoA acyltransferase1 (3-ketoacyl-CoA thiolase, ACAA1) antibodies directed against peroxisomal matrix proteins (Fig. 2). Gaussian-fitted line scan analysis showed the peaks of matrix proteins centered between the peaks of the peroxisome membrane profiles (Fig. 2C,F). Next, we analyzed the structure of hyper-tubulated peroxisomes induced by overexpression of PEX11β. PEX11β-myc expression in HeLa cells and immunostaining against the myc tag showed hyper-tubulated peroxisomal membranes in most of the cells 24 hours after transfection (Fig. 3A). STED microscopy revealed that these structures have a mean diameter of 91.8 nm ± 20.6 nm (±SD) (Fig. 3B,D). In addition, we found vesicular peroxisomal structures that appeared to undergo fission (Fig. 3C). Hyper-tubulated peroxisomes, with a length above 800 nm, were only evident in PEX11-induced conditions (Fig. 3E). This analysis shows that PEX11-induced peroxisome expansion is not associated with a change in peroxisome diameter.

### DLP1 and MFF localization on human peroxisomes at the nanoscale

**Figure 4:**
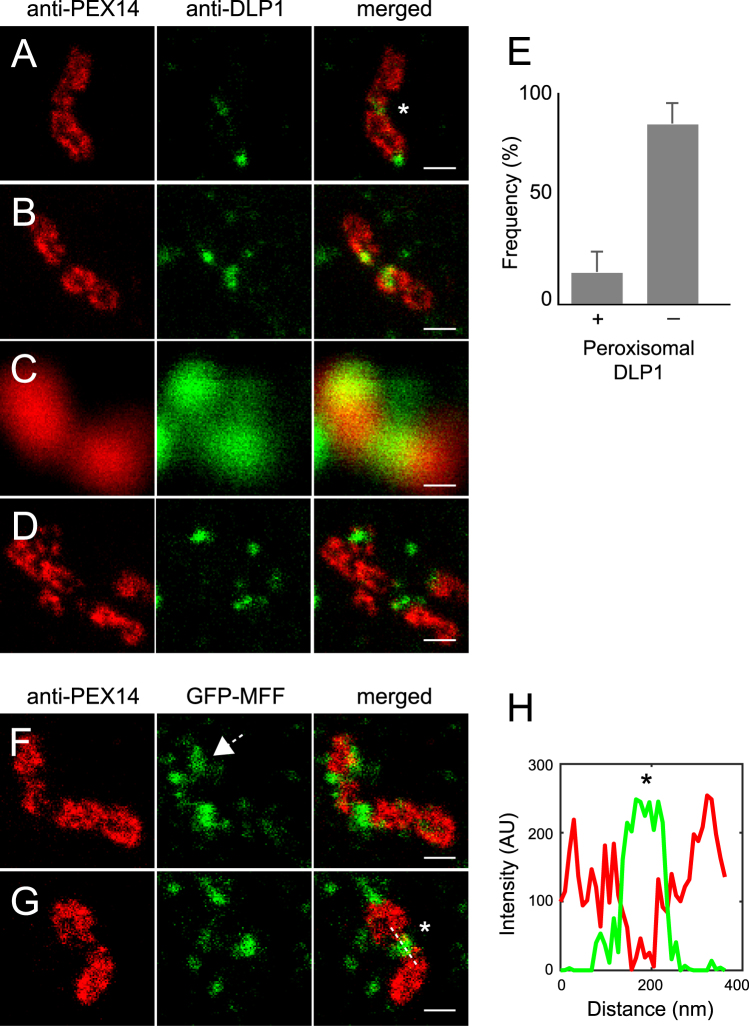
Localization of DLP1 and MFF at peroxisomal membranes by STED nanoscopy. (**A**,**B**) Dual immunofluorescence with anti-PEX14 labeled with KK114 secondary (red channel, left) and anti-DLP1 labeled with Atto594 secondary (green channel, middle) on human skin fibroblasts. (**C**) Confocal image of PEX14 and DLP1. (**D**) STED scan of the image in (**C**). (**E**) Object-based image analysis of DLP1 localization on peroxisomal membrane and free DLP1 puncta. DLP1 puncta (N = 212 peroxisomes) are analyzed from 8 optical sheets (corresponding to 8 independent cells randomly chosen by widefield mikroscopy). (**F**,**G**) HeLa cells transfected with GFP-MFF, and labelled with anti-PEX14/KK114 (red channel, left) and anti-GFP/Atto594 (green channel, middle). Asterisks indicate division sites. Arrow in (**F**) indicates a putative MFF half ring structure at a constriction. (**H**) Line scan plot of the line scan marked in (**G**). All images are STED or confocal raw data, linearly scaled for intensity. Scale bar 200 nm.

Peroxisomes proliferate by division employing a fission machinery that is shared with mitochondria^44^. We studied the arrangement of two important fission proteins, DLP1 and MFF with super-resolution microscopy. It was previously shown, that DLP1 is able to form helical rings around constricted mitochondria and endoplasmic reticulum membranes^30^. We used anti-DLP1 antibody to visualize endogenous DLP1 in human skin fibroblasts. Mitochondria were labeled with antibodies binding to the outer membrane protein TOM20. By this approach, we identified DLP1 puncta along the mitochondrial membrane, together with the more rare ring structures and half-ring structures on constricted and invaginated mitochondrial membranes (Supplementary Figure S1). When analyzing peroxisomes, we found constricted peroxisomal membranes carrying DLP1 puncta that appeared not to be part of ring structures (Fig. 4A). Further, DLP1 was associated with peroxisome membrane tips (Fig. 4B). It has previously been observed with diffraction-limited microscopy that DLP1 localizes to peroxisome tips and constriction sites of elongated peroxisomes^45^. However, diffraction-limited microscopy can easily provide misleading information about the localization of DLP1, not allowing the distinction between the peroxisomal and other DLP1 populations. The confocal image in Fig. 4C shows DLP1 apparently located at the constriction or fission hotspots of the peroxisome. Using STED we had to classify these DLP1 puncta as not associated with the peroxisome (Fig. 4D). Taking advantage of this increase in resolution, we quantified DLP1 puncta associated with peroxisomes by scoring an overlap of DLP1 with PEX14 as association with peroxisomes. The majority of DLP1 is not directly associated with peroxisomes (85%) and only a smaller portion (15%) is associated with peroxisomes (Fig. 4E). To complement the image analysis of peroxisomes undergoing fission, we visualized the MFF fission protein using GFP-MFF and an anti-GFP antibody. Diffraction-limited imaging showed MFF accumulation along peroxisome structures devoid of membrane proteins^29,46^. We identified sub-diffraction structures of GFP-MFF marking membrane division sites of peroxisomes (Fig. 4F–H). These results show the substantial improvement of fission factor resolution at peroxisomal membranes, which is offered by STED nanoscopy.

### Quantitative super-resolution analysis of ghost peroxisomes in ZSS patient fibroblasts

The description of residual membrane structures (ghosts) in ZSS patient cells by electron microscopy and indirect immunofluorescence microscopy has been important for connecting the genetic defect and the cellular phenotype^22,35,38,39,47^. However, it is not clear if differences in the structures of ghost membranes throughout the complementation groups and type of mutations can reveal information about the nature of the biogenetic defect along the path of peroxisome formation. We hypothesized that a robust quantitative imaging approach and an appropriate metric may contribute to this goal. We used STED microscopy and automated imaging analysis to analyze the size and morphology of peroxisomal ghosts in ZSS patient fibroblasts from different complementation groups.

ZSS patient fibroblasts were obtained from patients with typical clinical profile and with typical laboratory findings pointing to a ZSS disorder. A skin biopsy was performed and primary human fibroblasts were cultivated for every single patient. To identify the *PEX* gene affected in the individual patient, cells were transfected with different expression vectors containing one of all the so far known human *PEX* genes and then sequenced for the *PEX* gene that complements the defect as described previously^33^. Together with the biochemical and molecular data, clinical symptoms and disease course of the individual patient was recorded. All patients exhibited the well-known dysmorphic features for ZSS patients including big large fontanel, hypertelorism, epicanthus, broad nasal bridge, as well a typical biochemical profile with elevated levels of VLCFA, elevated levels of phytanic and pristanic acid as well as reduced levels of plasmalogens in whole erythrocyte lipids, hepatomegaly with elevated levels of transaminases, as well as core neurological features like muscular hypotonia, and failure to thrive. One of the patients described for the first time in this study was the PEX12 deficient patient. She showed an almost normal development till the age of four years with a rapid neurological deterioration afterwards, which was exceptional for ZSS patients. The other two newly described patients from this study the PEX5 deficient and PEX10 deficient patients (Table 1), showed a characteristic clinical course in line with the patients described previously.

**Table 1:** Overview of genetic, clinical and sub-diffraction phenotypes of patient fibroblasts.

PEX gene	Mutation	Catalase localization	Ghost size (rel. to wt)	PMP70 abundance (±SD) (rel. to wt)	Peroxisome number per cell (rel. to wt)	Clinical phenotype
*PEX1* ^−/−^	G843D	CYTO	2.0	1.07 ± 0.39	~24%	Mild^55,61^
*PEX1* ^−/−^	I700fsX42	CYTO	1.57	0.84 ± 0.25	~40%	Severe^61^
*PEX6* ^−/−^	S232HfsX15	CYTO	1.86	n.d.	~30%	Intermediate^50^
*PEX13* ^−/−^	W313G	CYTO	1.65	0.62 ± 0.16	~66%	Mild^50^
*PEX10* ^−/−^	L272fs	CYTO	1.53	0.47 ± 0.06	~23%	Severe *(this study)*
*PEX2* ^−/−^	F278LfsX3	CYTO	1.42	0.39 ± 0.02	~20%	Severe^50^
*PEX5* ^−/−^	Q133X	CYTO	1.25	n.d.	~43%	Severe *(this study)*
*PEX12* ^*−/*+^	L123del	CYTO/PX	1.19	0.65 ± 0.04	~72%	Mild *(this study)*
Control	—	PX	1	1	100%	Healthy

CYTO cytoplasmic; PX peroxisomal. The *PEX12*^*−/+*^ human skin fibroblasts present with a heterogeneous/mosaic catalase staining pattern with some cells showing peroxisomal localization of catalase and some with Zellweger-like cytoplasmic localization. Clinical phenotype: Severe (<1 year survival after birth), Intermediate (>1 year), Mild (>2 years). n.d. not determined.

**Figure 5:**
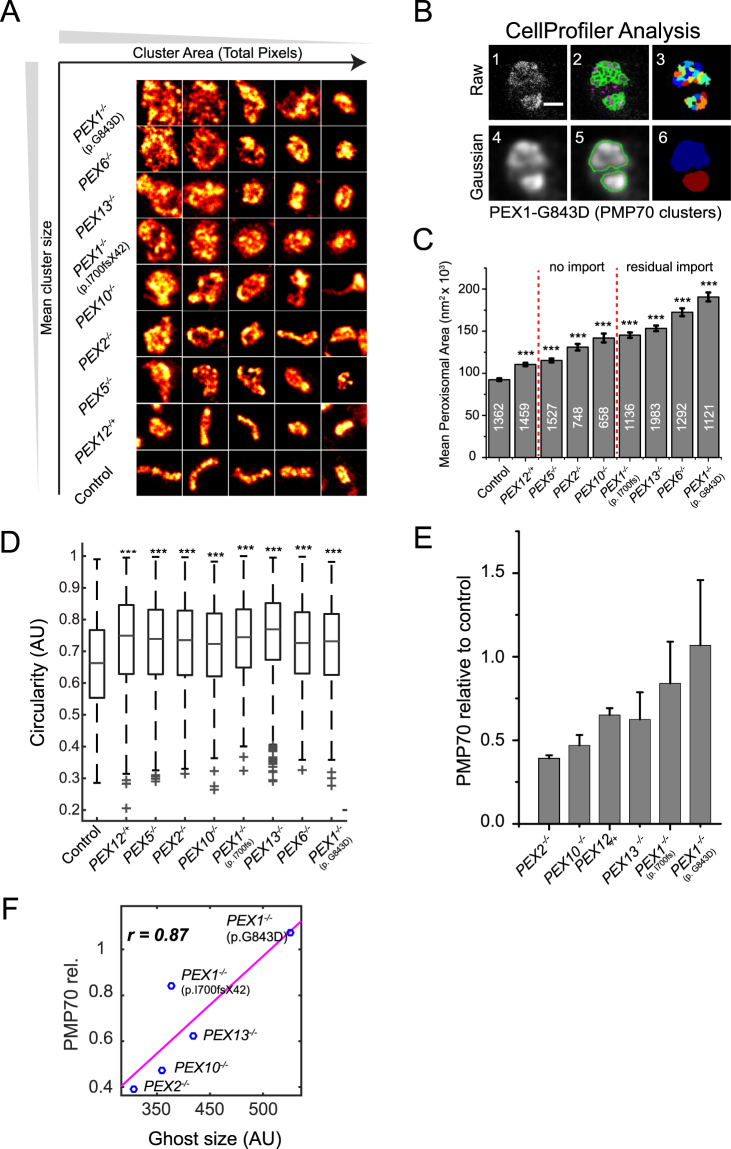
ZSS patient peroxisomal ghost analysis. (**A**) STED tiles (1000 × 1000 nm) of peroxisomal membranes form patient fibroblasts immunostained with anti-PMP70 and secondary antibody conjugated to Atto594. Tile images were smoothed with a 3 × 3 filter. (**B**) CellProfiler analysis workflow: (1) STED image of ghost peroxisomes, (2) segmentation of raw image, (3) object map of segmented antibody clusters, (4) Gaussian filter with 100 nm diameter applied, (5) segmentation results, (6) object map of segmented ghost peroxisomes. Scale bar 500 nm. (**C**) Gaussian analysis with 100 nm diameter. Bar graph indicates mean size of the peroxisomal structures. Ghost sizes were analyzed from three independent experiments, except *PEX6*^−/−^ and *PEX12*^*−/+*^ (N = 2). The number of ghost peroxisomes measured per condition is indicated on bars, and statistics relative to control peroxisome was calculated using the Kolmogorov-Smirnov test. ***p < 0.0001. Error bars: SEM. (**D**) Circularity (F-Circularity) of peroxisomal structures. PBD is associated with an increase in peroxisome circularity. ***p < 0.0001. Error bars: SD. (**E**) Quantitative Western blot analysis of PMP70 abundance shows reduced PMP70 in *PEX2*^−/−^ patient fibroblasts. N = 3 to 5. (**F**) Pearson correlation of PMP70 levels with ghost size of five homozygous ZSS patient fibroblasts cell lines showed positive correlation (p = 0.0541).

Using skin fibroblasts from the patients listed in Table 1, peroxisome ghosts were stained with anti-PMP70. Single ghosts were identified if the thresholded signals (local maxima) were localized within a diameter of 100 nm (Fig. 5A). Proximate ghost structures were separated by shape parameters (*CellProfiler*) and the surface areas of more than 600 ghost peroxisomes were analyzed per condition (Fig. 5B). Ghosts in ZSS patient cells are significantly larger than wild-type peroxisomes in control cell lines (Fig. 5C). STED microscopy and breaking the diffraction limit proved essential to discriminate the peroxisomal structures (Supplementary Figure S2A), because when we emulated a confocal setting by increasing the Gaussian diameter to 250 nm the differences in phenotypes changed and the overall ghost size increased due to the enlarged signal point spread function (PSF) and segmentation errors of proximate ghost structures (Supplementary Figure S2B).

The genetic mutations of the patients included in this study and the effect of mutations on residual import have been previously investigated^48^. PBD patients with severe homozygous mutations like p.I700fsX42 lack import activity of matrix proteins, while patients with milder mutations have detectable levels of residual import activity. By arranging patients according to increasing average peroxisomal size, we found that peroxisomal ghost size was lower in patients with severe mutations compared to the milder clinical phenotypes (Fig. 5C). *PEX1*^−/−^, *PEX6*^−/−^, and *PEX13*^−/−^ patients’ peroxisomes grouped on one end of the scale, while patients with mutation in *RING* peroxins and human control cells were found to display the smallest ghost size. The two *PEX1*^−/−^ patients fibroblasts showed a difference in their peroxisome size (Supplementary Figure S3). The more severely affected mutant form (p.I700fsX42) showed a significantly smaller average ghost size, whereas the less severely affected mutant with detectable and residually active PEX1 protein (p.G843D) showed enlarged ghost structures (Supplementary Figure S3). This is in accordance with the observation that a residual matrix protein import function of *PEX1*^−/−^ correlates with PEX1 abundance^49^. RING family ZSS patients and the *PEX5*^−/−^ patient (p.Q133X) presented here, all suffer severe defects that lead to loss of peroxisomal function and complete block of matrix protein import^50^. The *PEX6*^−/−^ patient showed a similar ghost phenotype as *PEX1*^−/−^ and *PEX13*^−/−^ patients, in agreement with the notion that the commonly mutated AAA-type ATPase *PEX* genes are able to partially complement each other^51^. Moreover, the docking factor *PEX13*^−/−^ (p.W313G) patient has been shown to have normal import of PTS2 proteins^52^. In *PEX13*^−/−^ patient fibroblasts we found ghost structures with luminal ACAA1 content (Supplementary Figure S4A) and also complex structures accumulating ACAA1 (Supplementary Figure S4B), which are reminiscent of the membrane systems that have been reported to be biogenetic intermediates^47^.

This quantitative analysis also revealed that the ghosts in ZSS cells appear more circular (less elongated) when compared to control cells (Fig. 5D). Based on this observation, we speculate that the de-tubulated, more circular structures of ghosts and their loss of membrane curvature functionally correlates with the impaired division of these abnormal structures^53^.

Previously, differences in PMP70 protein abundance in liver biopsies from ZSS patients were reported, suggesting that peroxisomal integral membrane proteins abundance are critical for the ghost size^36,41,42^. To investigate whether there is a correlation between the integral membrane proteins abundance and ghost size, we quantitated PMP70 by Western blotting in whole-cell lysates from ZSS patient fibroblasts with mutations in *PEX1*^−/−^ (p.G843D, p.I700fsX42), *PEX13*^−/−^ (p.W313G), *PEX10*^−/−^ (p.L272fs), *PEX2*^−/−^ (p.F278LfsX3), and *PEX12*^−/+^ (p.L123del) as well as from human control fibroblasts. Interestingly, the *PEX2*^−/−^ patient cells exhibited the lowest PMP70 abundance (Fig. 5E). When we arranged the homozygous ZSS patients according to increasing PMP70 abundance, we observed a positive relationship with the ghost size (Fig. 5F). Moreover, we quantified the peroxisome number per cells, and found that the number of peroxisomes was reduced in all ZSS fibroblasts, except for *PEX12*^*−/+*^ and *PEX5*^−/−^ mutated cell lines showing less reduction in their ghost abundance (Supplementary Figure S5A). However, correlation analysis did not indicate a correlation of ghost number with PMP70 protein abundance (Supplementary Figure S5B), which supports the hypothesis that not the ghost number but rather the ghost size is related to PMP70 abundance.

**Figure 6:**
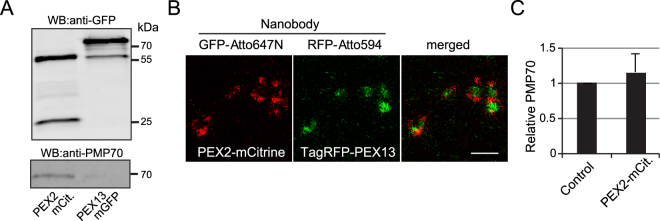
PEX2 interacts with PMP70. (**A**) Co-immunoprecipitation of HeLa cells transfected with PEX2-mCitrine or PEX13-mGFP. Cell lysate was immunoprecipitated with anti-GFP nanobody. The immunoprecipitate were analyzed by anti-GFP and anti-PMP70 antibodies. IP, immunoprecipitation; WB, Western Blot. (**B**) STED raw images of HeLa cells co-transfected with PEX2-mCitrine and TagRFP-PEX13 immunolabeled with GFP and RFP nanobodies conjugated to Atto647N and Atto594, respectively. Scale bar 500 nm. (**C**) Quantitative Western blot analysis of PMP70 abundance in HeLa cell lysates transfected with GFP empty vector and PEX2-mCitirine. Differences were not statistically significant as measured by one-way ANOVA: p < 0.05. N = 3.

We further investigated the significantly reduced PMP70 abundance in a *PEX2*-deficient patient. We used immunoprecipitation to pull down PEX2-mCitrine by nanobodies and found a PEX2-PMP70 interaction that could explain the strong reduction in PMP70 protein abundance in this ZSS patient’s cells (Fig. 6A). We overexpressed PEX2-mCitrine and PEX13-mGFP (negative control) in *PEX2-* and *PEX13*-deficient ZSS patient cells, respectively. Both PEX2-mCitrine and PEX13-GFP were able to restore biogenesis as depicted by catalase import in the respective PBD conditions (Supplementary Figure S6), which indicate normal protein function of our tagged proteins. We also did not see change in PMP70 abundance under PEX2 overexpression (Fig. 6B). Therefore, differences in PMP70 protein abundance are most likely secondary to translocon assembly defects. Integral PMPs abundance might have a direct effect on peroxisomal membrane formation. However, the function of PMP70 does not support any membrane remodeling effect of PMP70. We also tested the colocalization of PEX2-mCitirine and TagRFP-PEX13 with catalase marker (Supplementary Figure S7). Intriguingly, STED imaging of PEX2-mCitirine and TagRFP-PEX13 showed segregation of the two peroxins on distinct membrane compartments (Fig. 6C and Supplementary Figure S8). Altogether, our results confirm that different degrees of severity of ZSS, which correlate with residual import function, are associated with distinctive morphological ghost phenotypes (Table 1).

## Discussion

Recently it was shown that super-resolution microscopy could resolve the peroxisomal membrane and matrix compartment in human skin fibroblasts transfected with a PTS1 reporter protein and co-stained for PEX14 (ref.^43^). The average size of the peroxisome was determined to be ~350 nm^43^. We applied super-resolution STED imaging to study peroxisomes in non-transfected mammalian cells and found a lower average diameter of ~100 nm. The earlier reported larger peroxisomal diameter is possibly due to overexpression of the reporter matrix protein leading to organelle enlargement and/or due to differences in growth conditions between these studies. A 100 nm peroxisome diameter in fibroblasts matches size measurements by electron microscopy in fibroblasts and ranges at the lower limit of what has been found in hepatocytes^52,53^. Our two-color STED approach shows that it is possible to localize and analyze the distribution of endogenous matrix and membrane proteins at nanometer resolution. The subdiffraction structure of PEX11-induced peroxisomes is in agreement with previous electron microscopy images showing elongated (hyper-tubulated) structures with no apparent change in the peroxisomal membrane diameter^28^. Furthermore, STED microscopy proved to give new insights for the peroxisomal localization of the fission factors DLP1 and MFF.

We quantitatively studied the residual membrane structures (ghosts)^34,35,38^ in human skin fibroblasts by STED microscopy and found that larger and more complex ghost structures are present in those patients known to have residual import of matrix proteins and consequently better prognosis^1,48,52^. The *PEX1* gene is the most commonly affected gene in ZSS^54,55^. Patients bearing the PEX1 p.G843D mutation tend to have the mildest ZSS phenotype, while patients with p.I700fsX42 (c.2097_2098insT) are more severely affected. STED analyses for a patient with p.G843D mutation revealed enlarged complex ghost structures, whereas the patient with p.I700fsX42 mutation showed small ghost sizes. This is in accordance with the observation that the residual matrix protein import in *PEX1*^−/−^ cells correlates with residual PEX1 abundance and a milder clinical course of disease^49^. These results were confirmed in the mildly affected *PEX13*^−/−^ patient as well as in the severely affected *PEX2*^−/−^, *PEX5*^−/−^ and *PEX10*^−/−^ patients. In a previous study, the *PEX13*^−/−^ patient has been shown to have normal import of PTS2 proteins^52^. In accordance, we found complex ghost structures accumulating ACAA1, which are reminiscent of the membrane systems that have been reported to be biogenetic intermediates^47^. Recent studies point to a strong and unanticipated association of peroxisome biogenesis with autophagy^21,56,57^, and it is a plausible hypothesis that autophagy rather than impaired fission of ghost peroxisomes is at the basis of this phenotype. This may also explain the differences in PMP70 abundance in those patients with smaller ghost peroxisomes and is in agreement with the hypothesis that variability of ghosts could be caused by variable PMP70 protein levels^35,58^. *PEX2*^−/−^ ZSS cells have lower PMP70 abundance relative to wild-type control. These findings could reflect the recently identified enzyme-substrate relationship of the E3-ligase function of PEX2 and PMP70 (ref.^14^) In order to elucidate the mechanism underlying the ghost phenotype and peroxisome biogenesis it will be interesting to analyze the peroxisome translocon complex together with other PMPs by single molecule imaging,

In conclusion, the resolution advance delivered by STED microscopy allowed us to characterize in more detail normal human peroxisome morphology and its defects in ZSS. We localized the fission proteins DLP1 and MFF to peroxisomal membranes with unprecedented detail and highlighted the characteristics of the hyper-tubulated peroxisomes upon PEX11β overexpression. In ZSS patients, quantitative STED analysis and classification of the peroxisome remnants revealed distinct morphological phenotypes of ghost peroxisomes so that a quantitative connection between sub-diffraction morphology and clinical phenotype can be drawn.

## Patients, Materials and Methods

### Patients

Eight ZSS skin fibroblast cell cultures from seven different complementation groups (*PEX1, PEX2, PEX5, PEX6, PEX10, PEX12 and PEX13*) were included in the study. The diagnosis was established by characteristic clinical and molecular findings^33,50^. We obtained written informed consent from guardians of all patients. Experiments with human fibroblasts were approved by the ethics committee of the University Medical Center Göttingen and were conducted in accordance with the relevant guidelines and regulations.

### DNA cloning and plasmids

PEX13-mGFP and PEX2-mCitrine have been described previously^52^. *PEX13* was PCR-amplified from human cDNA and cloned into HindIII and BamHI restriction sites of TagRFP-C (Evrogen). *PEX11* was PCR-amplified from human cDNA and cloned into NheI and NotI sites of pcDNA3.1*myc*/His (−) (Invitrogen). Clones were checked by restriction digestion and DNA sequencing using the BigDye kit (Applied Biosystems) and analyzed by a sequencer chromatograph analyzer (Applied Biosystems).

### Cell culture, transfection and immunofluorescence

Control and ZSS patient fibroblasts were cultured in low glucose Dulbecco’s Modified Eagle Medium (DMEM) medium (Biochrom GmbH, Germany) supplemented with 1% Pen/Strep (100units/ml Penicillin and 100 µg/ml Streptomycin), 1% (w/v) glutamine and 10% (v/v) Fetal Calf Serum (FCS) in 5% CO_2_ at 37 °C. For all experiments cells were detached with Accutase® (Life Technologies), washed once with PBS Dulbecco (Biochrom GmbH, Germany), and counted using Neubauer hemocytometer, according to manufacturer protocol. Equal densities of cells for all conditions and cell lines were seeded and cell culture conditions were kept constant to ensure maximal reproducibility. In rescue experiments, cells were transfected using Effectene transfection reagent (Qiagen, UK). Medium with transfection reagent was changed after 6 to 8 hours and cells were incubated for a total of 24 hours before being used in downstream experiments. For immunofluorescent detection of peroxisomal catalase, we followed the same protocol as previously described^33^. Primary antibody: Rabbit anti-catalase (Oxisresearch 24316, 1:400), secondary antibody: donkey anti-rabbit IgG conjugated to Cy3 (Jackson ImmunoResearch, 1:400). For STED experiments cells were fixed using 10% formaldehyde freshly prepared from 37% stock, blocked in 10% bovine serum albumin (BSA) in PBS, and permeabilized using 0.5% Triton-100× in PBS. The following primary antibodies were used: monoclonal mouse anti- PMP70 (Sigma SAB4200181, 1:500), polyclonal rabbit anti-PEX14 (Proteintech 10594-1-AP, 1:500), polyclonal rabbit anti-catalase (1:500), polyclonal rabbit anti-ACAA1 (Proteintech 12319-2-AP, 1:500), monoclonal mouse anti-DLP1 (BD Biosciences, 1:100), monoclonal mouse anti-GFP (Invitrogen living colors JL-8, 1:1000), monoclonal mouse anti-Myc (Cell Signaling 9B11, 1:1000). Primary antibodies were incubated in PBS with 1% BSA for 1 hour at 37 °C. The following secondary antibodies were used: Sheep anti-mouse immunoglobulin (Dianova) coupled to Atto594 (1:500, Atto-TEC) or KK114 (1:50, Abberior). Secondary antibodies were added in 1% BSA in PBS for 1 hour at 37 °C. Cells were washed three times in PBS between each antibody incubation and mounted on Mowiol mounting medium containing DABCO. HeLa cells were cultured in low glucose DMEM similar to human skin fibroblasts in 5% CO_2_ at 37 °C and trypsin was used to detach adherent cells. Single transfection experiments were done using Effectene; for co-transfection we used LipofectamineLTX PLUS (Thermofisher). For nanobody staining we used GFP-Atto647N (Chromotek gba-647N) and RFP-Atto594 (Chromotek rba-594) nanobody boosters at 1:50 dilution.

### Widefield microscopy

Widefield images were obtained using the 100× oil objective (1.3 NA) with a Zeiss Imager M1 fluorescence wide field scope equipped with the Zeiss Axiocam HRm Camera and Zeiss Axiovision 4.8 acquisition software. ImageJ software (NIH, USA) was used for linear contrast enhancement of image, cropping and scale bars. Images were arranged using Adobe Illustrator software.

### STED microscopy (nanoscopy)

We used a custom built, time-gated STED (gSTED) setup for single as well as two color imaging as reported previously^59^. For the RFP/GFP co-transfection experiments with nanobody labeling we used a commercial two color STED setup (Abberior Instruments). Both setups use a pulsed STED laser at 775 nm wavelength, a 595 nm laser to excite Atto590 and 594 dyes, and a 640 nm laser to excite Atto647N and KK114 dyes. The excitation laser powers were optimized on single-color samples for minimal color channel crosstalk^59^.The Imspector acquisition software (by Andreas Schönle, Max Planck Institute for Biophysical Chemistry, Göttingen, Germany, available through Max-Planck-Innovation GmbH, Munich, Germany) was used on both setups and acquisition parameters were kept constant for quantitative measurements. For figure preparation, raw data images were linearly scaled in ImageJ and arranged with Adobe Illustrator.

### Sub-diffraction size analysis

Raw data were handled in ImageJ, smoothed with σ = 1 pixel Gaussian function and line scans were obtained using line drawing tool in ImageJ through structures of interest. Line scan data were copied into Matlab (www.mathworks.com) and processed for data fitting (Gaussian function) using a custom-made automated routine. Histogram and boxplot diagrams and statistics were produced and calculated in Matlab. To measure peroxisome length, peroxisome structures were straightened using ImageJ plug-in. The length of the straightened peroxisome structures was measured manually using length measurement tools in ImageJ.

### PEX11β hyper-tubulation analysis

To measure peroxisome length induced by PEX11β overexpression, images were blurred with a Gaussian filter (σ = 30 nm) and IsoData threshold algorithm was applied to blurred images to facilitate individual peroxisome segmentation. Binary peroxisome objects were then skeletonized using the “skeletonize” plug-in in ImageJ and to measure skeleton length, we used the “AnalyzeSkeleton” plug-in (ImageJ, NIH). Data were analyzed and plotted using Matlab (www.mathworks.com). Peroxisomes stained with anti-PEX14 in non-transfected HeLa cultures served as controls.

### Automated image analysis

Images were analyzed using a *CellProfiler* (www.cellprofiler.org) pipeline designed based on previously published method^60^. Raw data images were smoothed using average 3 × 3 ImageJ smooth function to eliminate background. Gaussian blurring with a diameter of 100 nm was applied to images in *CellProfiler*, in order to aid the identification of single ghost peroxisomes rather than identifying antibody clusters (over segmentation artifact). Blurred images were divided into 50 × 50 pixels blocks and adaptive Maximum Correlation Threshold (MCT) algorithm (lower limit: *0.05*, upper limit *1*) was computed for each block. Shape and local maxima were used to distinguish boarders, identify, and declump adjoining ghost peroxisomes. Holes within identified objects were filled after thresholding and declumping. To count individual antibody clusters within peroxisomal structures, raw data images were thresholded by computing two class global Otsu thresholding (upper limit: *0.12*, lower limit: *1*) with automatic smoothing settings provided in *CellProfiler*. Intensity and local maxima were used to identify and declump adjoining clusters. Relating clusters before and after Gaussian using Relate object module allowed us to identify children (antibody clusters) per each parent (peroxisome structure). Only ghost peroxisome parents with children objects above zero were included in the downstream analysis. Circularity (F-circularity) was determined by dividing the minor axis length by the major axis. The more elongated the peroxisomes the lower is the circularity factor. All analysis parameters were implemented in an automated *CellProfiler* pipeline to ensure unbiased analysis. Boxplot, bar graphs and statistics tests were done using OriginPro software and figures were arranged in Adobe Illustrator. Analyses were done on at least three independent experiments, or as indicated in the figure legends.

### DLP1 object based analysis

Background was measured in raw STED data images in non-stained areas and the maximum background intensity was subtracted in ImageJ using the math tool. Images were then linearly scaled and a Gaussian filter of 10 nm (sigma) was applied to reduce pixels’ intensity variabilities, to reduce over segmentation errors. Similar algorithms to the one explained earlier were implemented in *CellProfiler* to select individual peroxisomes. For DLP1 puncta selection, a Gaussian blur of 30 nm diameter was used to select for individual DLP1 structures. Next, an object based colocalization analysis was carried out in *CellProflier* using its built-in “relate object module”. Free DLP1 are puncta that show no overlap with peroxisomal marker (anti-PEX14), and objects (DLP1) overlapping with PEX14 are counted as peroxisomal DLP1. Data was analyzed and plotted in Excel to calculate averages and standard deviation. The data is represents eight independent cells randomly chosen by widefield microscopy.

### Ghost peroxisome quantification

Images were acquired by the M1 Imager wide-field microscope with 100× oil objective (semi-automated analysis). Images were saved in ImageJ as color images. Color images were separated into respective channels in *CellProfiler* and named accordingly. The nuclei and peroxisomes were found and segmented by a primary object identification module, whereas cell borders (semi-automated analysis) were manually defined with the manual free drawing object module. Peroxisome signal was thresholded using Otsu threshold and segmented by intensity automatic settings. Nuclei were smoothed with Gaussian filter prior to segmentation to remove nuclei speckles and improve segmentation efficiency. Finally, a relate object module was implemented to quantify peroxisome number per cell. Data analysis was done using OriginPro statistics software. Figures were arranged using Adobe Illustrator.

### Quantitative Western blot analysis

Cell lysates were prepared in radioimmunoprecipitation assay (RIPA) buffer (20 mM Tris-HCl, pH 7.4, 150 mM NaCl, 2 mM EDTA, 1% NP40, 1 mM DTT, 0.1 mM PMSF, Complete protease inhibitor [Roche, Switzerland]). For quantitative Western blot total protein was measured using BCA assay kit (Roche) and equal proteins amounts were loaded for each condition. Proteins were separated in 12% SDS-PAGE gels and blotted using semi-dry technique. Membrane blots were blocked in 5% Milk Buffer in PBST (PBS, 0.1% Tween20). To increase quantitative output membranes were cut in two strips, probed simultaneously for anti-PMP70 (Sigma SAB4200181, 1:1000) and anti-GAPDH loading control (Abcam ab8245, 1:5000) in 1% milk powder in PBST buffer overnight at 4 °C with horizontal shaking. After washing with PBST, we added HRP-conjugated secondary monoclonal donkey anti-mouse antibody (1:5000) at  room temperature for 1 hour. Protein bands were detected by immersing in LumiLight (Roche) for 3 min. Images were scanned using chemiluminescent image system ImageQuant LAS-4000 (GE Healthcare). Exposure times were kept minimal to avoid saturation. Protein bands were quantified using Image Studio software (LI-COR). PMP70 signal intensities were normalized to GAPDH.

### Co-immunoprecipitation

Immunoprecipitation of mCitrine and GFP fusion proteins was done using GFP-TrapA nanobody coupled to agarose beads (Chromotek, Germany) following the manufacturer’s protocol. Cells lysate were prepared in RIPA buffer and the second beads wash following the binding was done in higher salt stringency buffer (500 mM NaCl instead of 150 mM). One tenth of lysate volume of bound and unbound fractions were loaded onto 12% SDS-PAGE gels, blotted onto nitrocellulose membranes, and probed with mouse monoclonal anti-PMP70 (1:500), mouse monoclonal anti-ALDP (BD Bioscience 2D-ALD6, 1:500), polyclonal rabbit anti-PEX14 (1:500), or rabbit polyclonal anti-GFP (Abcam Ab290, 1:2000). HRP-conjugated monoclonal donkey anti-mouse antibody (1:5000) and polyclonal donkey anti-rabbit antibody (1:5000) were used to detect probed proteins. Protein bands were detected using LumiLight PLUS and scanned by LAS4000 analyzer.

### Data availability

The raw data generated during and/or analyzed during the current study are available from the corresponding author on reasonable request.

## Electronic supplementary material

Supplementary information
