# Supplementary material for: Super-resolution imaging reveals the sub-diffraction phenotype of Zellweger Syndrome ghosts and wild-type peroxisomes
Source: Sci Rep. 2018 May 17;8:7809. doi: 10.1038/s41598-018-24119-2 (PMC5958128; doi:10.1038/s41598-018-24119-2)
Supplement: Supplementary file 1 — Supplementary information [file 41598_2018_24119_MOESM1_ESM.pdf]

Supplementary Material

Super-resolution imaging reveals the sub-diffraction phenotype of Zellweger Syndrome ghosts and wild-type peroxisomes

Kareem Soliman[1,3], Fabian Göttfert[2], Hendrik Rosewich[1], Sven Thoms[1*], Jutta Gärtner[1]

[1] Department of Pediatrics and Adolescent Medicine, University Medical Center Göttingen, Georg August University Göttingen, Robert-Koch-Strasse 40, 37075 Göttingen, Germany

[2] Department of NanoBiophotonics, Max Planck Institute for Biophysical Chemistry, Am Faßberg 11, 37077 Göttingen, Germany

[3] Current Address: College of Pharmacy, Arab Academy for Science, Technology and Maritime Transport, Abou Keer, P.O. Box 1029, Alexandria, Egypt

* Corresponding Author: Sven Thoms, sven.thoms@med.uni-goettingen.de

Supplementary Figures

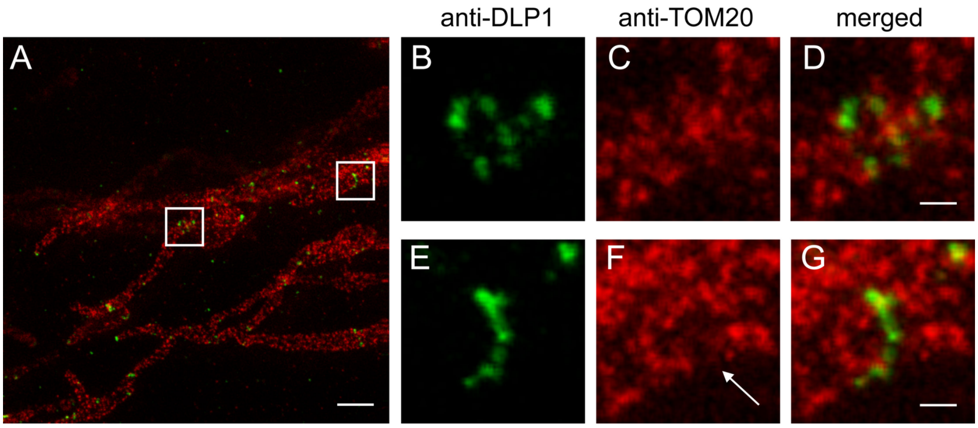

Figure S1. Mitochondrial DLP1 structures on constricted and invaginated membranes. Human skin fibroblasts immunostained with anti-DLP1 and anti-TOM20, labeled with Atto594- and KK114 labeled secondary antibodies, respectively. (A) STED image overview of one cell. Scale bar 1 µm. (B-G) Blow-up of regions marked in (A). DLP1 stain in B shows potential circular structure surrounding mitochondrial outer membrane. Arrow in (F): Constricted mitochondrial membrane with DLP1 half-ring marking mitochondrial membrane invagination. Scale bar 200 nm.

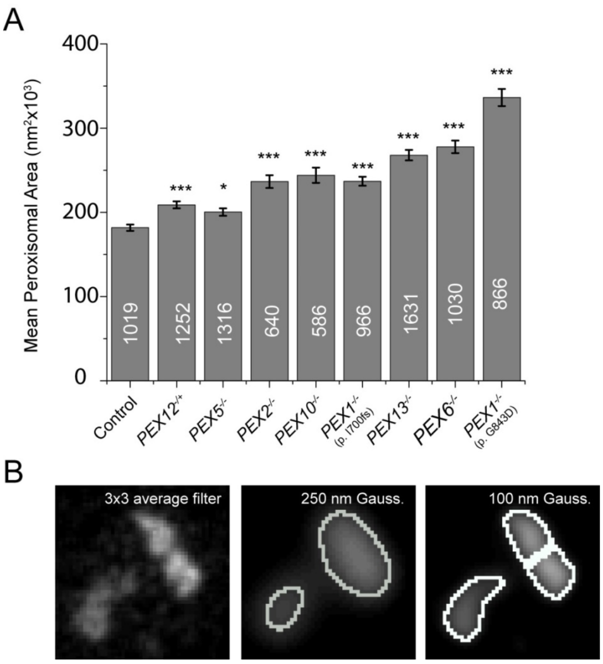

Figure S2. Ghost analysis with Gaussian filter. (A) Fibroblasts from healthy controls and patients were stained with anti-PMP70 antibodies and secondary antibodies conjugated to Atto594. STED images were blurred using a Gaussian filter (250 nm). Bar graph indicates mean peroxisomal size. Statistics significance relative to control: *p<0.01, **p<0.001, and ***p<0.0001. (B) Example of smoothened STED image by 3x3 average filter and segmentation results of wild-type peroxisomes using various Gaussian filters. Only at 100 nm filtering, distinct peroxisomal structures are accurately recognized.

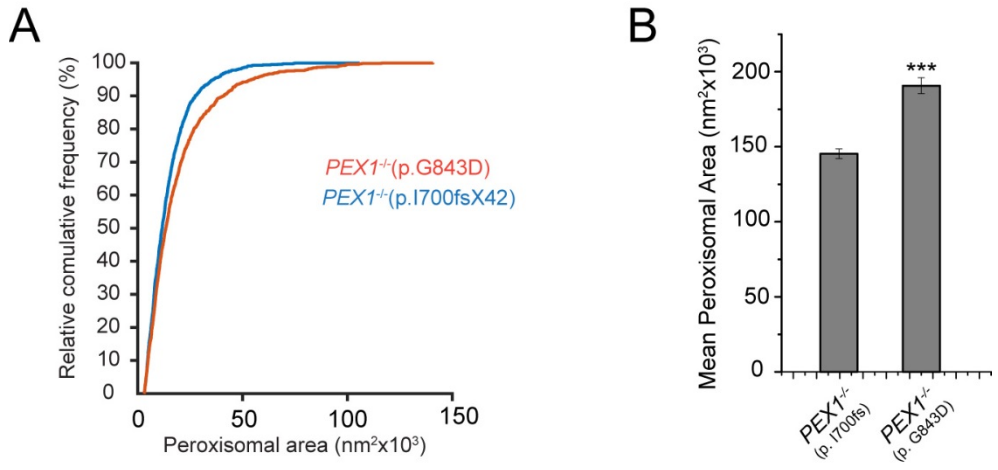

Figure S3. Comparison of PEX1-G843D and PEX1-I700fsX42. (A) Culmulative frequency of the peroxisomal area of PEX1-G843D and PEX1-I700fsX42 fibroblasts. (B) Mean peroxisomal area of the patients' peroxisomes in (A). p-value < 0.0001 relative to PEX1-I700fsX42, K-S test. Error bars show ±SEM.

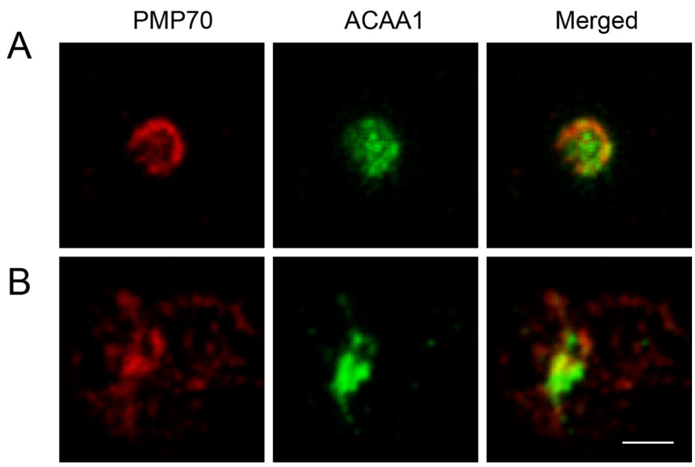

Figure S4. STED imaging of ACAA1 ghost content in *PEX13*$^{-/-}$ patient fibroblasts. Immunofluorescence with anti-PMP70 (red) and anti-ACAA1 (green). (A) Circular peroxisome ghost with ACAA1-filled lumen. (B) Complex peroxisome ghost structure with accumulation of ACAA1 in the center. Scale bar 500 nm (for A and B).

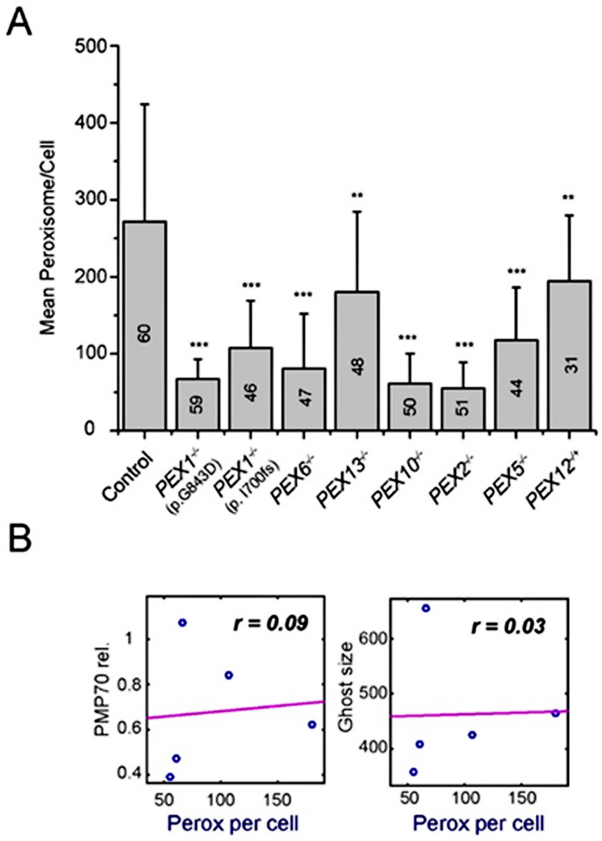

Figure S5. Ghost particles quantification in ZSS patient fibroblasts and control cells. (A) Semi-automated analysis of number of ghost particles immunostained for PMP70. Error bars show SD. Significance levels (t-test) of peroxisome size are given relative to control: $^{**}p<0.001$ and $^{***}p<0.0001$. (B) Absence of correlation of peroxisome abundance with PMP70 levels and with ghost size.

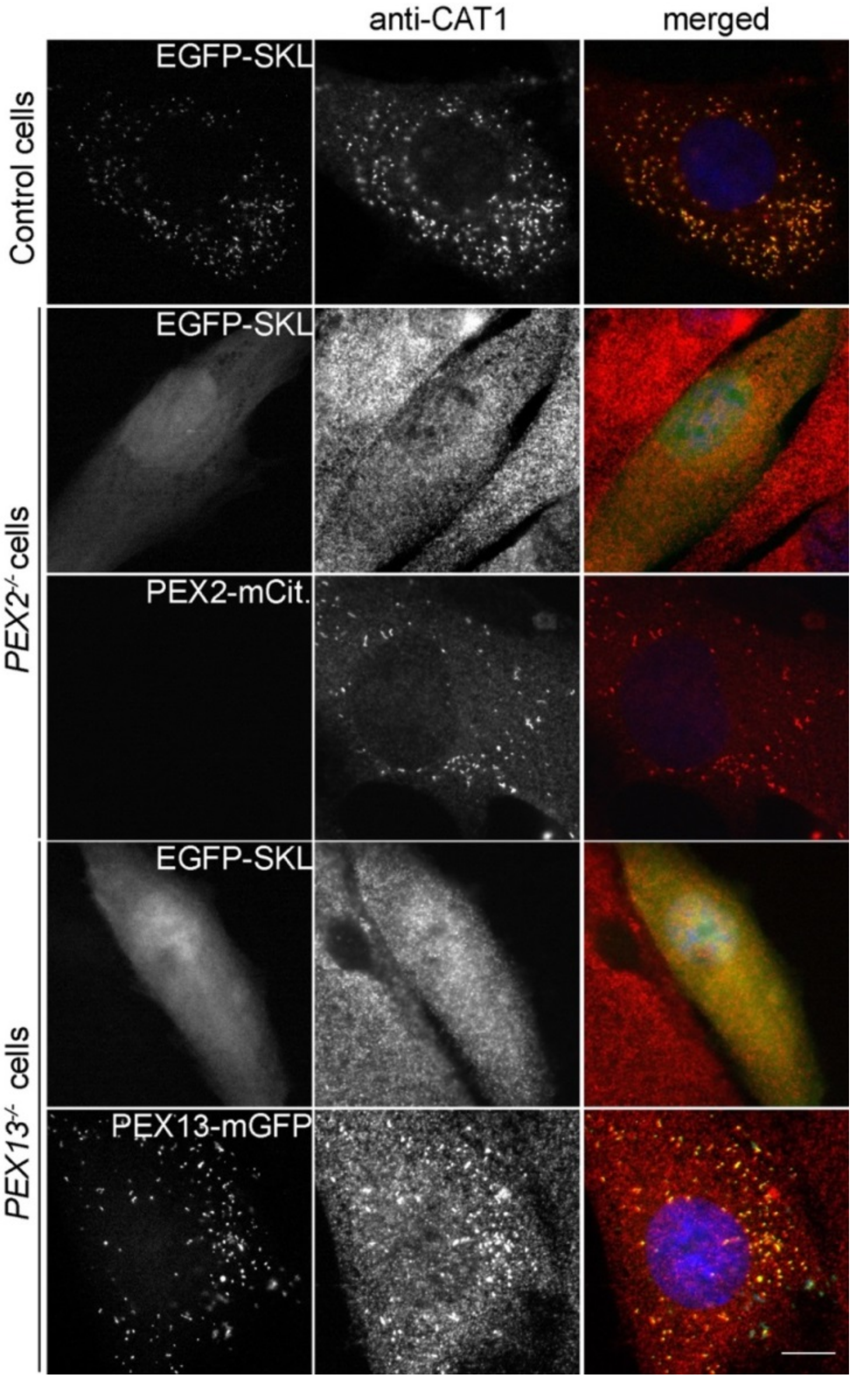

Figure S6. Peroxin fluorescent fusion-protein complementation. Control and ZSS patient cells were transfected with peroxisomal fusion proteins and processed for immunofluorescence catalase using anti-catalase (anti-CAT1) antibodies. PEX2-mCit and PEX13-mGFP can rescue peroxisome formation in ZSS human skin fibroblasts. Scale bar 10 µm.

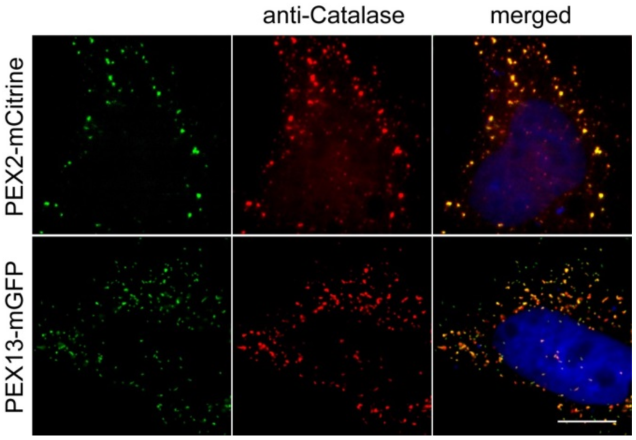

Figure S7. Peroxisomal localization of fluorescently labeled peroxins.
Peroxin fusion proteins transiently expressed in HeLa cells for 24 hours. Cells were fixed and processed for immunofluorescence using anti-CAT1 (catalase) antibody labeled by donkey anti-rabbit Cy3 secondary antibody. Scale bar 10 µm.

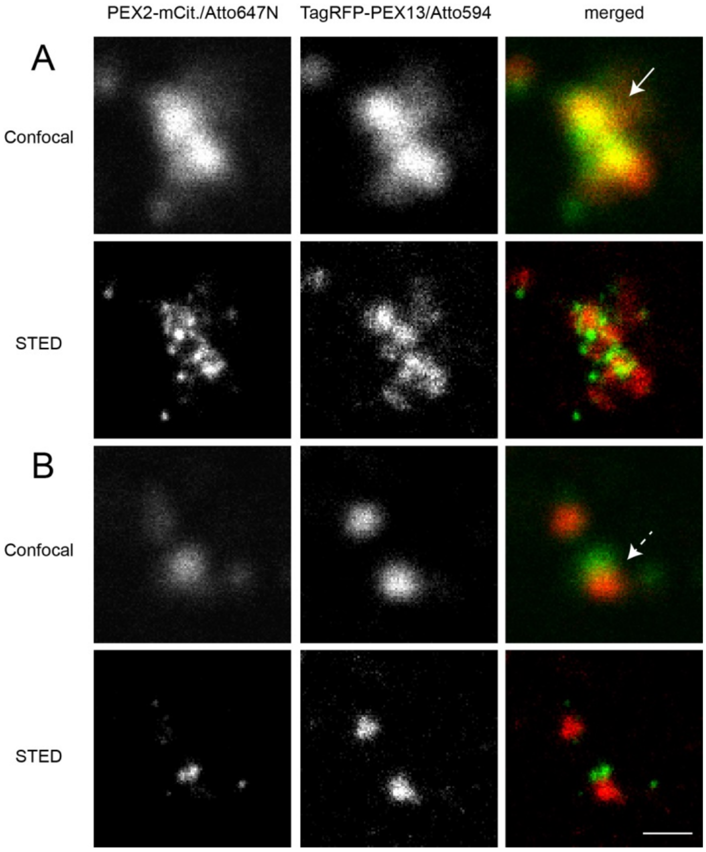

Figure S8. STED imaging shows PEX2 and PEX13 segregate on distinct membrane compartments. (A) Confocal images showing co-localized PEX13 and PEX2, while the STED image shows the two proteins segregate on two different membrane compartments. (B) Confocal image and STED images showing vesicular staining of PEX2 and PEX13, no colocalization detected in both confocal and STED settings. HeLa cell co-transfected with PEX2-mCitirine and TagRFP-PEX13. PEX2-mCitirine labeled with ATTO647N nanobody and TagRFP-PEX13 labeled with Atto594-nanobody. Scale bar 500 nm.
